# Decline in Sexual Risk Behaviours among Young People in Zambia (2000–2009): Do Neighbourhood Contextual Effects Play a Role?

**DOI:** 10.1371/journal.pone.0064881

**Published:** 2013-05-23

**Authors:** Nkomba Kayeyi, Knut Fylkesnes, Nora Wiium, Ingvild F. Sandøy

**Affiliations:** 1 Centre for International Health, University of Bergen, Bergen, Norway; 2 Department of Public Health, School of Medicine, University of Zambia, Lusaka, Zambia; 3 Department of Psychosocial Science, University of Bergen, Bergen, Norway; University of Washington, United States of America

## Abstract

**Objective:**

This study examined trends in premarital sex, multiple partnership and condom use among young people (15–24 years) in Zambia from 2000 to 2009, and assessed the effects of individual and neighbourhood variables on these sexual behaviour indicators in 2000 and 2009.

**Methodology:**

We analysed data from the Zambia Sexual Behaviour Survey, conducted in 2000, 2003, 2005 and 2009. Multi-stage cluster sampling was used to select 385 neighbourhoods, giving a population sample of 6,500 young people. Using linear-by-linear trend test, trends in the three indicators were examined. Multilevel logistic regression was used to assess the effects of individual and neighbourhood variables on the indicators.

**Results:**

Premarital sex among young people decreased significantly from 51 to 42% between 2000 and 2009. Multiple partnerships of men also decreased from 26 to 14% during the same period. The use of condoms by young people remained stable during this period. Full multilevel regression models explained 29 and 34% of the neighbourhood variance of premarital sex in 2000 and 2009. For multiple partnerships and condom use, the explained variance was 29 and 18% in 2000; whereas in 2009 it was extremely low. Urban residence and living in neighbourhood with higher average duration of residence were associated with low premarital sex and higher condom use. Living in a neighbourhood with higher average level of comprehensive knowledge of HIV was associated with less risky sexual behaviour.

**Conclusion:**

Declining trends in premarital sex and multiple partnerships are among the factors that might explain the decrease in HIV incidence in Zambia among young people. However, condom use among young people has remained low and stable over the years. The results also suggest that behaviour change interventions should take stock of the social context when introducing individual-level programmes because neighbourhood factors play a considerable role in influencing sexual behaviour.

## Introduction

The Joint United Nations Programme on HIV/AIDS (UNAIDS) finally acknowledged in its 2010 report that the HIV incidence had declined by >25% between 2001 and 2009 in 22 sub-Saharan African countries [1]. The strongest evidence for the decline in Zambia is from the sentinel HIV surveillance of pregnant women, repeated population-based surveys in selected communities, and the 2001/2 and 2007 Zambia Demographic and Health Surveys (ZDHS) [2], [3], [4]. National trend data on HIV prevalence among men are lacking. However, repeated population surveys in selected communities in Zambia showed that HIV trends among men were decreasing at almost the same rate as women [3]. These declines in HIV incidence have been mainly attributed to positive change in sexual behaviour [1], [5], [6]. The repeated population-based study in selected communities found parallel declines in HIV prevalence and number of sexual partners in the same sub-groups for both young men and women [5].

According to the UNAIDS 2010 report, young people (15–24 years) accounted for 46% of all new infections in Zambia, with an estimated 120,000 young men and women currently being HIV positive [1], [7]. After the 2001 United Nations General Assembly Special Session (UNGASS), Zambia renewed its interest in monitoring sexual risk behaviour indicators among young men and women [8]. To date, Zambia has reported twice (in 2010 and 2012) to UNGASS on the progress made towards the targets set for HIV/AIDS in 2001 (i.e to reduce sexual transmission of HIV by 50% by 2015– by increasing condom use in high risk groups to 80% and increasing comprehensive knowledge of HIV among young people to 95%) [8], [9]. Seven indicators relating to HIV/AIDS among young people have generally been monitored by UNAIDS, based on previous validation. These indicators include median age at sexual debut, premarital sex, condom use at last premarital sex, number of partners in the previous year, condom use at last higher risk sex, condom use at first sex, and age mixing in sexual relationships. Our study focused on only 3 of these factors, namely premarital sex, multiple partnerships and condom use at last premarital sex [10], [11], since national data for these indicators were available for the period 2000–2009. In order to provide effective planning and delivery of HIV prevention programmes, understanding trends in risky sexual behaviour and what factors influence them, is important.

Most previous studies on factors affecting sexual risk behaviours have been restricted to individual-level factors [5], [12], [13], [14], [15], [16], [17], [18], but more recent evidence suggests that neighbourhood-level factors also play an independent and significant role in shaping behaviour [15], [19], [20], [21], [22], [23], [24]. Only a few studies have explored the effect of neighbourhood-level factors on HIV infection in Zambia [24], [25], [26], [27]. One of these studies from Ndola[27], examined the effects of different measures of neighbourhood socio-economic status on HIV infection among young women in Zambia. Another study from Chelston and Kapiri Mposhi [26] examined the average neighbourhood educational attainment on HIV infection among young women. Both these studies found that neighbourhood characteristics were significantly associated with the risk of HIV infection.

In this study, we used data from the 4 latest rounds of the Zambia Sexual Behaviour Surveys (ZSBS) to examine trends in premarital sex, multiple partnerships and condom use at last premarital sex in different subgroups of young people. The effects of individual and neighbourhood-level factors on the 3 sexual risk behaviour indicators among young people were also assessed.

## Methods

### Settings

Zambia is a landlocked country located in Southern Africa and has an estimated population of 13 million people [28], of which young people of ages 15 to 24 years make up 21.5% of the population. The HIV prevalence in Zambia is estimated to be 14.3% among adults 15–49 years of age [29], with the current prevalence among young men and women being 4.2 and 8.9%, respectively [1].

### Data collection procedure

The Sexual Behaviour Survey (ZSBS) is a nationally representative population based cross-sectional survey that has been conducted in Zambia in 1998, 2000, 2003, 2005 and 2009, and has collected data on HIV/AIDS/STIs knowledge, attitudes, sexual behaviour, and health-care seeking behaviours [30], [31], [32], [33], [34]. This data has been used to monitor national indicators proposed by international programmes, such as the Millennium Development Goals (MDGs), UNAIDS, the President's Emergency Plan for AIDS Relief (PEPFAR), UNICEF, and the Global Fund to Fight AIDS, Tuberculosis and Malaria. However, the indicators monitored have been evolving with time and this has resulted in changes in the questions posed by the ZSBS. For example, the initial questionnaire used in the 1998 survey was based on the World Health Organisation (WHO)/Global Programme on AIDS (GPA) prevention indicators and the Family Health International (FHI) general population surveillance questionnaire [30]. In 2000, an updated questionnaire was made with a new set of standards and indicators developed by an international consortium led by UNAIDS [31]. Over the years, adjustments have been made to the 2000 questionnaire to accommodate new indicators monitored by the international community. Although changes have been made to the questionnaires over the years, the core information on the indicators of interest for this study have remained the same (except for employment) [34]. But since the 1998 questionnaire lacked some of the standard questions included in the later surveys, it was excluded from the present analysis.

Ethical approval for the ZSBS was granted by the University of Zambia Ethics Committee. All eligible participants were informed of the purpose of the survey, and both written and oral consent to participate in the survey was sought. Selected participants were given the option of accepting or declining the interview. For participants <18 years, a parent or guardian was also asked for permission to interview them. This study was anonymous, and confidentiality was assured in all the 4-rounds of the surveys [30], [31], [32], [33], [34].

### Sampling Procedure

A 2-stage probability random sampling procedure was used in all 4-rounds of the ZSBS. The first stage of the sampling involved the selection of urban and rural clusters in every province of Zambia. These clusters, which we used as proxies for neighbourhoods, are census tracts or standard enumeration areas (SEAs), with an average size of 130 households or 600 people [29]. In the 2000 survey, a total of 80 clusters were sampled from the sampling frame of 312 clusters of the 1996 Zambia Demographic and Health Survey (ZDHS). The number of clusters was increased to 100 in the 2003 survey, drawn from the 2000 Population and Housing Census as a sampling frame. The 2005 survey drew 105 clusters and the 2009 survey drew 100 clusters from the 2001/2 ZDHS sampling frame. In the period under review, a total of 385 clusters were selected in the 4 surveys and the distribution of these clusters between urban and rural areas was proportional to the national distribution of urban and rural residents [30], [31], [32], [33], [34].

The second stage of the sampling process involved the selection of households in the sampled clusters. About 16 households (20 households in 2000) were sampled per urban cluster and 34 households (30 households in 2000) were sampled per rural cluster. A higher number of households were sampled in rural clusters because rural households on average have fewer adult members. This resulted in a total number of sampled households to 1,851 in 2000, 2,497 in 2003, 2,465 in 2005, and 2,500 in 2009. All females aged 15–49 years and males aged 15–59 years in the selected households were eligible to participate in the survey.

### Variables

Since the design of the ZSBS was not based on a theoretical model, the selection of variables to include in the analyses of this paper was based on an extensive review of published articles that have examined sexual behaviour [19], 20,21,35,36,37,38. The operational definitions and response categories of the selected variables for the analyses are presented in Table S1.

#### Dependent variables

Three dependent variables considered for this study were: premarital sex, multiple partnership and condom use at last premarital sex. Premarital sex was defined as the number of young people who reported sexual intercourse in the last 12 months among all single young people [11]. This variable was derived from the question that asked if young people had ever had sex, but excluded all those who reported that they had sex for the first time when they were living with a partner and those who reported to be married, divorced or widowed. Young people who were not formally married were therefore excluded if they were living together with a partner, since a man and a woman living together for >3 months in Zambia are recognised as being married by customary laws, despite the absence of a marriage license. Multiple partnership was defined as the number of young people who had sex with more than one partner in the past 12 months among all sexually active young people, irrespective of whether these partners were concurrent or not [11]. Condom use at last premarital sex was defined as reporting use of condoms, and the denominator was young unmarried sexually active people, i.e. excluding those who were widowed or divorced [11].

#### Individual independent variables

The following independent individual variables were analyzed: sex, age, marital status, educational attainment, employment status, religion and urban/rural residence (definitions in Table S1). It is noteworthy that there was a change in the question concerning employment status between the 2003 and 2005 surveys. In the 2000 and 2003 surveys, the respondents were asked “*what kind of work they mainly did*” or “*their occupation*”. Those who mentioned specific work or occupation were categorised as “*employed*”, whereas students, family workers without pay, retirees, housewives and respondents who reported that they were not working were categorised as “*unemployed*”. In the 2005 and 2009 surveys, respondents were asked whether they were *employed or not*, and those responding positively were categorised as “*employed*” and those not employed as “*unemployed*”.

#### Neighbourhood predictor variables

Variables describing characteristics of the neighbourhoods were created by aggregating individual responses within each cluster for all respondents aged 15–59 years [39]. Neighbourhood educational attainment was derived by aggregating individual-level years of school attendance. Neighbourhood labour force participation in 2000 and 2003 was based on the proportion of individuals who were categorised as employed on the question regarding their main kind of work done or their occupation, whereas in the 2005 and 2009 rounds, neighbourhood labour force participation was based on the proportion of individuals reporting that they were employed. As a proxy of social cohesion in the neighbourhood - an aspect of social capital, we created a variable that we called ‘neighbourhood residential stability’ by aggregating the number of years the participants had lived in the same neighbourhood. Neighbourhood comprehensive knowledge of HIV was based on the average number of correct responses made by respondents to the 5 questions presented in Table S1.

### Analyses

All the analyses were restricted to young people (15–24 years). Descriptive statistics for the population distribution and variables are shown in Table S2, S3, S4 & S5. Trend analyses of the sexual risk behaviour indicators against the individual and neighbourhood characteristics, stratified by gender, were conducted using a Chi-square linear-by-linear trend test. In the trend analyses, the continuous neighbourhood variables were arbitrarily categorized into 3 levels: neighbourhoods with the lowest 40%, the middle 40% and the highest 20% [38].

Multilevel mixed effects logistic regression *(xtmelogit)* on the 3 sexual risk behaviour indicators were analysed using STATA 11.1 (StataCorp, 2009). All neighbourhood variables were analysed as continuous variables, but were standardised before running the multilevel analysis. The multilevel models tested included the following:

1. a random intercept-only model (model 1)
2. a random intercept model including individual-level variables only (model 2)
3. a random intercept model including neighbourhood-level variables only (model 3)
4. a random intercept model including both individual and neighbourhood level variables (model 4).

These model tests were run for the 3 dependent variables using 2000 and 2009 data. Interaction tests were conducted for individual and neighbourhood variables, but none was found to be statistically significant. Other multilevel statistics estimated included the explained variance (R^2^), the unexplained variance at the neighbourhood-level (or intraclass correlation (ICC)) and log-likelihood tests. To estimate the explained variance of the models, we initially considered the formula suggested by Raudenbush and Bryk (2002) [40]. Using this formula, a negative explained variance was found after adding individual variables to the intercept-only model in 2000 for multiple partnerships and in 2009 for premarital sex. Raudenbush and Bryk's formula assumes that the sampling procedure used to obtain samples at all levels was simple random sampling, as such when the predictor variables are added to the models that had more group-level variance than the simple random sampling process produced, the apparent within-group variance increased. This produced a negative estimate for the explained variance at the lower level [40]. To avert the negative explained variance problem, Hox [40] suggested a formula proposed by Snijders and Bosker [41] and it defined the explained variance (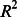) in a multilevel logistics regression model as: 
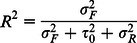



Where: 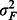 is the explained part of the total variance,


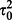
 is the unexplained variance at the neighbourhood-level, and


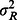
 is the unexplained variance at the individual-level (assumed π^2^/3 = 3.29).

In the logistic distribution for the level-one residual, the variance is assumed to be a constant (π^2^/3 = 3.29) [41], which implies that the degree at which individuals belonging to the same neighbourhoods resemble each other. The intraclass correlation coefficient (ICC) of a 2-level logistic intercept-only model with an intercept variance of 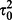 is expressed as:
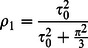



The ICC at the neighbourhood-level is defined as the proportion of total variance between neighbourhoods [42], which is the same as the unexplained neighbourhood-level variance (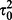). The models were checked by goodness-of-fit test using the log-likelihood test. We also used the log-likelihood test to check whether adding covariates to the intercept-only model significantly improved the model fit. Associations with p<0.10 were taken as significant.

## Results

### Population sample and response rate

In total 8,687 females and 7,803 males were interviewed between 2000 and 2009. The response rates were 88% in 2000, 87% in 2003, 88% in 2005, and 97% in 2009 among females, and 85% in 2000, 85% in 2003, 86% in 2005, and 88% in 2009 among males [30], [31], [32], [33], [34]. However, the focus of this study was young people aged 15–24 years, who totalled 6,500 participants in the years under review, i.e. 1376 (2000), 1835 (2003), 1695 (2005) and 1594 (2009).

### Descriptive statistics

In all the surveys, there were slightly more young women (54–60%) than young men who were interviewed. The majority had never married (61–73%) and lived in urban areas (60–64%). The relative distribution of participants by gender, age, religion, urban/rural residence was relatively stable across the surveys. However, the proportion with secondary or higher education increased with time from 38 to 51%, as did the mean neighbourhood residential stability (from 11.9 to 12.6 years) and comprehensive knowledge of HIV (from 12% to 48%). The “proportion employed” in 2000 and 2003 was substantially higher than the “proportion employed” according to the definition applied in 2005 and 2009. Similarly, neighbourhood labour force participation estimates in 2000 and 2003 were much higher than the estimates in 2005 and 2009 (Table S2).

### Premarital sex

There was a 17% decrease in premarital sex among young women (from 47 to 39%) and men (from 54 to 45%) from 2000 to 2009 (Table 1). The decline in premarital sex of young women was particularly marked among participants aged 15–19 years, those with low education, rural residents, and those residing in neighbourhoods with high educational attainment, low and medium residential stability and neighbourhoods with high comprehensive knowledge. However, an increasing trend in premarital sex was observed among young protestant Christian women. For young men, significant declines in premarital sex were seen irrespective of individual educational attainment or urban-rural residence and among those aged 15–19 years, protestant Christians, and those residing in neighbourhoods with medium educational attainment, low and medium residential stability, and low and medium comprehensive knowledge (Table 1).

**Table 1:** Premarital sex trends among young people (15–24 years) by gender and by survey year (percentage).

		Female					Male				
		2000	2003	2005	2009	*p*	2000	2003	2005	2009	*p*
**Dependent variable**											
Premarital sex		46.8	48.2	40.2	38.9	***0.003***	54.4	57.5	52.5	45.1	***<0.001***
**Independent variables**											
***Individual variables***											
Age at last birthday											
	15–19	36.8	40.5	31.1	28.7	***0.004***	41.9	44.5	36.0	29.6	***<0.001***
	20–24	73.0	70.2	65.3	65.9	*0.201*	80.4	77.5	77.9	75.8	*0.344*
Highest level of school attended											
	None/Primary	45.9	44.9	44.3	33.0	***0.006***	50.4	54.8	53.6	36.4	***<0.001***
	Secondary/Higher	47.8	51.2	36.2	43.2	*0.106*	59.6	60.4	51.7	51.5	***0.015***
Employment											
	Not employed	37.2	41.9	34.5	34.6	*0.228*	44.2	49.4	45.2	40.6	*0.071*
	Employed	66.2	55.6	68.0	64.8	*0.863*	67.9	65.8	75.9	68.9	*0.466*
Religion											
	Catholic Christians	37.6	47.4	40.2	34.0	*0.336*	53.0	60.3	55.9	46.5	*0.149*
	Protestant Christians	50.5	51.6	59.8	59.9	***0.004***	55.8	56.5	51.9	44.6	***<0.001***
Residence											
	Rural	51.3	50.4	44.4	41.1	***0.013***	58.8	57.2	54.9	46.6	***0.001***
	Urban	42.6	45.9	35.8	36.5	*0.066*	49.2	57.9	49.0	42.9	***0.023***
***Neighbourhood variables***											
Educational attainment											
	Low	46.4	45.1	31.6	39.5	*0.058*	48.8	54.6	51.2	47.0	*0.371*
	Medium	49.3	53.1	48.1	42.4	*0.104*	59.7	61.4	53.8	41.0	***<0.001***
	High	41.8	46.9	42.4	28.0	***0.045***	59.4	56.5	52.6	50.0	*0.176*
Labour force participation											
	Low	42.2	47.9	41.6	35.3	*0.062*	49.8	57.7	53.1	45.4	*0.092*
	Medium	54.5	47.3	37.8	43.1	***0.039***	59.2	55.1	52.8	47.0	***0.010***
	High	46.7	51.3	42.2	37.5	*0.140*	57.7	62.1	49.4	40.3	***0.002***
Residential stability											
	Low	47.5	52.1	39.5	40.6	***0.039***	56.9	61.1	53.1	46.5	***0.003***
	Medium	49.3	45.4	44.2	37.2	***0.018***	52.5	53.6	50.6	42.6	***0.010***
	High	35.4	43.2	29.6	38.3	*0.946*	52.4	58.1	56.1	48.2	*0.319*
Comprehensive knowledge											
	Low	54.5	51.9	47.0	43.5	***0.030***	55.7	61.0	61.3	46.3	***0.012***
	Medium	43.1	45.8	33.5	36.1	***0.057***	53.8	54.8	48.2	41.3	***0.001***
	High	39.2	46.6	40.8	36.9	*0.422*	53.0	56.5	45.5	49.7	*0.383*

***P = *** p-values of the chi^2^ linear-by-linear trend test; highlighted figures are significant at p<0.05

In Table 2, the intercept-only model for 2000 shows that premarital sex varied across neighbourhoods with a neighbourhood difference of 5%, which was statistically significant at p <0.05. The addition of individual-level variables in model-2 reduced the neighbourhood difference by ∼40%, but only explained 27% of the variance of premarital sex. In model-3, the inclusion of neighbourhood variables in the intercept model reduced the difference between neighbourhoods to 2%, but these variables explained only 9% of the variance in premarital sex. The inclusion of both neighbourhood and individual-level variables in model-4 explained about 29% of the variance in premarital sex, while the difference between neighbourhoods was 2%, which was not statistically significant. The log-likelihood test showed that the addition of both individual and neighbourhood variables to the intercept-only model (model-4), significantly improved the fit. Model-4 also showed a number of statistically significant associations with background factors: older age, employment and being a protestant Christian were associated with higher odds of premarital sex, whereas residing in an urban area or neighbourhoods with high residential stability and high comprehensive knowledge of HIV were associated with lower odds of premarital sex among young people (Table 2).

**Table 2:** Multilevel logistic regression models for the premarital sex indicator among young people aged 15–24 years in Zambia, in 2000 and 2009.

Dependent variables		Pre-marital sex 2000				Pre-marital sex 2009			
		Multivariate				Multivariate			
		Model 1	Model 2	Model 3	Model 4	Model 1	Model 2	Model 3	Model 4
			AOR	AOR	AOR		AOR	AOR	AOR
**Fixed effects**		(95% Cl)	(95% Cl)	(95% Cl)	(95% Cl)	(95% Cl)	(95% Cl)	(95% Cl)	(95% Cl)
***Individual variables***									
Sex									
	Male		1.00		1.00		1.00		1.00
	Female		0.83 (0.60–1.14)		0.82 (0.59–1.14)		0.89 (0.66–1.20)		0.89 (0.66–1.20)
Age			1.43 (1.32–1.55)^***^		1.43 (1.32–1.55)**		1.64 (1.52–1.76)^***^		1.65 (1.53–1.77)^***^
Highest level of school attended									
	None/Primary		1.00		1.00		1.00		1.00
	Secondary/Higher		1.20 (0.82–1.77)		1.14 (0.77–1.70)		1.29 (0.91–1.83)		1.28 (0.90–1.82)
Employment									
	Not employed		1.00		1.00		1.00		1.00
	Employed		1.82 (1.22–2.70)^**^		1.83 (1.20–2.79)^**^		2.06 (1.32–3.21)^**^		2.19 (1.40–343)^**^
Religion									
	Catholic Christians		1.00		1.00		1.00		1.00
	Protestant Christians		1.41 (0.96–2.06)^*^		1.40 (0.95–2.04)^*^		0.85 (0.57.1.27)		0.86 (0.57–1.28)
Residence									
	Rural		1.00		1.00		1.00		1.00
	Urban		0.58 (0.38–0.89)^**^		0.58 (0.34–1.01)^*^		0.42 (0.26–0.70)^**^		0.34 (0.18–0.65)^**^
***Neighbourhood variables^β^***									
	Educational attainment			0.98 (0.82–1.18)	1.02 (0.82–1.27)			0.96 (0.79–1.16)	0.86 (0.67–1.10)
	Labour force participation			1.21 (0.99–1.47)^*^	0.99 (0.72–1.36)			0.93 (0.76–1.14)	0.72 (0.55–0.93)^**^
	Residential stability			0.82 (0.69–0.98)^**^	0.79 (0.65–0.97)^**^			1.06 (0.87–1.28)	0.83 (0.62–1.11)
	Comprehensive knowledge			0.81 (0.70–0.95)^**^	0.82 (0.69–0.98)^**^			0.90 (0.73–1.10)	0.77 (0.59–1.00)^**^
**Fixed –effect model test**									
	Wald *x^2^*		128.86^**^	17.24^**^	133.79^**^		211.08^**^	2.07^**^	216.64^**^
	Degree of freedom		6	4	10		6	4	10
**Unexplained neighbourhood-level variance (SE)**		0.184 (0.10)^**^	0.146 (0.12)^**^	0.07 (0.07)	0.09 (0.10)	0.400 (0.13)^**^	0.783 (0.22)^**^	0.391 (0.13)^**^	0.657 (0.20)
**Model statistics**									
	Explained variance (R^2^)		0.270	0.089	0.288		0.330	4.59e–3	0.344
	Unexplained neighbourhood-level variance as	0.05	0.031	0.019	0.019	0.11	0.129	0.106	0.109
	a proportion of total variance								
	Unexplained individual-level variance as a		0.699	0.892	0.694		0.541	0.890	0.547
	proportion of total variance								
	Log likelihood	−548.74	−461.04	−571.44	−456.24	−760.22	−597.59	−774.48	−593.01
	Log likelihood test^δ^		<0.001	0.003	<0.001		<0.001	0.721	<0.001

Highlighted figures statistically significant at the following points: ^*^P<0.10; ^**^P<0.05; ^***^P<0.01; ^β^Neighbourhood variables interpreted as OR per unit increase; **^δ^**Tests whether adding individual and neighbourhood variables to model-1 significantly improves the fit of the model (P<0.05); Abbreviations:R^2^, explained variance; ICC, inter-class correlation; Cl, confidence interval; AOR, adjusted odds ratio; SE, standard error

For 2009, the intercept-only model showed that premarital sex varied across neighbourhoods, with a neighbourhood difference of 11%, which was statistically significant at p <0.05. Model-2 indicates that individual variables were much stronger predictors of premarital sex, such that their addition increased the unexplained neighbourhood-level variance from a proportion of 0.40 to 0.78. The inclusion of these variables explained 33% of the variance in premarital sex. Model-3, with neighbourhood-level variables, had a very low neighbourhood variance of premarital sex among the neighbourhoods. In contrast, model-4, which included both neighbourhood and individual-level variables, explained ∼34% of the variance in premarital sex, but the difference between neighbourhoods remained at 11%. The log-likelihood test revealed that despite these results, the inclusion of both individual and neighbourhood variables significantly improved the fit. Model-4 also showed that 2 individual-level variables (age and being in employment) were significantly associated with higher odds of premarital sex among young people. Urban residence and residing in a neighbourhood with high labour force participation and high comprehensive knowledge of HIV gave significantly lower odds of reporting premarital sex (Table 2).

### Multiple sexual partners

There was no clear overall trend in multiple partnerships among young women between 2000 and 2009. However, a statistically significant decrease of ∼45% was generally observed among young men, and almost all subgroups showed statistically significant declining trends. The only subgroup of men where a decline could not be seen was among those living in neighbourhoods with high comprehensive knowledge of HIV, but the change was not statistically significant (Table 3).

**Table 3:** Multiple partner trends among young people (15–24 years) by gender and by survey year (percentage).

		Female					Male				
		2000	2003	2005	2009	*p*	2000	2003	2005	2009	*p*
**Dependent variable**											
Multiple partners		3.5	4.2	5.1	2.0	*0.274*	25.8	19.3	15.1	14.1	***<0.001***
**Independent variables**											
***Individual variables***											
Age at last birthday											
	15–19	4.6	3.8	7.1	2.9	*0.773*	26.4	19.8	18.3	13.6	***0.038***
	20–24	2.8	4.5	3.9	1.6	*0.265*	25.0	19.1	13.9	15.1	***0.012***
Ever married											
	Never	4.9	8.5	15.1	2.6	*0.570*	28.4	19.4	15.6	14.5	***0.002***
	Married	3.1	3.0	2.5	1.8	*0.261*	21.2	19.2	14.5	14.7	*0.179*
Highest level of school attended											
	None/Primary	3.0	4.1	4.1	1.5	*0.341*	20.8	17.8	16.1	11.9	***0.053***
	Secondary/Higher	4.8	4.6	7.5	2.7	*0.415*	33.3	21.7	13.5	16.8	***0.003***
Employment											
	Not employed	2.4	6.1	5.4	2.3	*0.568*	22.7	19.2	19.7	14.1	*0.097*
	Employed	4.1	3.9	4.3	1.4	*0.229*	26.7	19.4	10.4	15.5	***0.002***
Religion											
	Catholic Christians	4.2	4.2	1.9	2.9	*0.482*	23.2	10.5	7.8	13.3	*0.126*
	Protestant Christians	3.3	4.3	5.8	1.9	*0.349*	26.2	22.1	17.3	14.9	***0.003***
Residence											
	Rural	3.2	2.6	5.2	1.3	*0.390*	25.1	18.0	17.1	14.1	***0.010***
	Urban	3.9	7.8	4.4	3.3	*0.495*	26.6	22.3	9.8	15.7	***0.044***
***Neighbourhood variables***											
Educational attainment											
	Low	3.1	4.3	4.3	1.9	*0.486*	18.0	18.3	14.1	14.6	*0.415*
	Medium	3.2	4.3	5.8	1.6	*0.477*	31.6	16.8	17.3	17.0	***0.012***
	High	4.3	4.0	4.8	2.9	*0.644*	26.2	26.8	11.9	8.3	***0.003***
Labour force participation											
	Low	3.0	8.0	5.5	3.7	*0.814*	20.5	24.6	13.7	10.5	***0.011***
	Medium	2.9	2.3	4.7	1.1	*0.479*	29.5	20.0	16.7	14.3	***0.004***
	High	5.1	2.1	5.2	0.0	*0.106*	25.9	9.5	15.0	21.7	*0.849*
Residential stability											
	Low	3.1	6.8	4.3	2.4	*0.457*	27.6	24.3	15.8	21.0	*0.133*
	Medium	3.3	3.7	6.3	1.6	*0.508*	21.8	14.4	13.2	8.1	***0.005***
	High	4.3	0.0	4.0	2.2	*0.602*	30.0	17.6	18.3	15.8	*0.083*
Comprehensive knowledge											
	Low	2.7	4.5	7.8	2.6	*0.726*	32.3	23.5	23.1	17.0	***0.009***
	Medium	4.3	4.2	2.9	2.1	*0.197*	22.5	16.5	12.0	10.1	***0.009***
	High	3.8	3.7	3.0	0.0	*0.121*	13.5	13.4	3.3	16.0	*0.866*

***P = *** p-values of the chi^2^ linear-by-linear trend test; highlighted figures are significant at p<0.05

The intercept-only model in 2000 showed that multiple partnerships varied across neighbourhoods with a neighbourhood difference of 14%. The addition of individual variables in model-2 reduced the neighbourhood difference in multiple partnerships to 10%, whereas these variables explained ∼29% of the variance in multiple partnerships. Model-3 showed that the inclusion of neighbourhood variables explained ∼2% of the variance in multiple partnerships. The addition of both individual and neighbourhood variables in model-4 explained ∼29% of the variance in multiple partnerships, while the difference between neighbourhoods was reduced to 9%. Model-4 showed that the inclusion of both individual and neighbourhood variables significantly improved the fit. Being female was the covariate significantly associated with lower odds of multiple partnerships in model-4. However, having secondary/higher educational attainment and being in employment were significantly associated with higher odds of multiple partnerships among young people (Table 4).

**Table 4:** Multilevel logistic regression models for multiple partnerships among young people aged 15–24 years in Zambia, in 2000 and 2009.

Dependent variables		Multiple partnerships 2000				Multiple partnerships 2009			
		Multivariate				Multivariate			
		Model 1	Model 2	Model 3	Model 4	Model 1	Model 2	Model 3	Model 4
			AOR	AOR	AOR		AOR	AOR	AOR
**Fixed effects**		(95% Cl)	(95% Cl)	(95% Cl)	(95% Cl)	(95% Cl)	(95% Cl)	(95% Cl)	(95% Cl)
***Individual variables***									
Sex									
	Male		1.00		1.00		1.00		1.00
	Female		0.12 (0.06–0.23)^***^		0.12 (0.06–0.23)^**^		0.12 (0.05–0.28)^***^		0.12 (0.05–0.28)^***^
Age			0.98 (0.86–1.11)		0.98 (0.86–1.12)		0.97 (0.84–1.12)		0.98 (0.85–1.14)
Ever married									
	Never		1.00		1.00		1.00		1.00
	Married		0.56 (0.29–1.10)^*^		0.57 (0.29–1.12)		1.12 (0.47–2.65)		1.08 (0.45–2.59)
Highest level of school attended									
	None/Primary		1.00		1.00		1.00		1.00
	Secondary/Higher		2.01 (1.09–3.71)^**^		2.01 (1.08–3.73)^**^		1.50 (0.75–3.03)		1.50 (0.74–3.05)
Employment									
	Not employed		1.00		1.00		1.00		1.00
	Employed		2.17 (1.05–4.46)^**^		1.96 (0.92–4.16)^*^		1.01 (0.49–2.06)		0.97 (0.46–2.06)
Religion									
	Catholic Christians		1.00		1.00		1.00		1.00
	Protestant Christians		1.02 (0.51–2.02)		1.02 (0.51–2.02)		0.98 (0.43–2.24)		0.96 (0.41–2.22)
Residence									
	Rural		1.00		1.00		1.00		1.00
	Urban		1.12 (0.54–2.36)		1.31 (0.54–3.22)		1.24 (0.61–2.52)		1.49 (0.60–3.68)
***Neighbourhood variables^β^***									
	Educational attainment			1.09 (0.81–1.45)	1.24 (0.86–1.77)			0.83 (0.60–1.16)	0.94 (0.64–1.36)
	Labour force participation			0.99 (0.71–1.39)	0.97 (0.57–1.65)			0.94 (0.66–1.33)	0.97 (0.65–1.44)
	Residential stability			0.95 (0.72–1.25)	1.03 (0.73–1.44)			0.95 (0.69–1.31)	0.96 (0.65–1.44)
	Comprehensive knowledge			0.77 (0.57–1.04)^*^	0.83 (0.59–1.17)			0.73 (0.50–1.07)	0.69 (0.46–1.02)^*^
Fixed –effect model test									
	Wald *x^2^*		63.44^**^	3.47	64.97^**^		32.43^**^	3.34	35.65^**^
	Degree of freedom		7	4	11		7	4	11
**Unexplained neighbourhood-level variance (SE)**		0.376 (0.25)^**^	0.522 (0.36)^**^	0.250 (0.24)	0.457 (0.35)^**^	3.63e–18	1.22e–11	5.08e–12	5.08e–15
**Model statistics**									
	Explained variance (R^2^)		0.286	0.023	0.294				
	Unexplained neighbourhood-level variance as	0.14	0.098	0.069	0.086				
	a proportion of total variance								
	Unexplained individual-level variance as a		0.616	0.908	0.620				
	proportion of total variance								
	Log likelihood	−266.01	−201.06	−264.16	−199.70	−163.48	−142.60	−162.75	−140.65
	Log likelihood test^δ^		<0.001	0.482	<0.001		<0.001	0.490	<0.001

Highlighted figures statistically significant at the following points: ^*^P<0.10; ^**^P<0.05; ^***^P<0.01; ^β^Neighbourhood variables interpreted as OR per unit increase; **^δ^**Tests whether adding individual and neighbourhood variables to model-1 significantly improves the fit of the model (P<0.05); Abbreviations:R^2^, explained variance; ICC, inter-class correlation; Cl, confidence interval; AOR, adjusted odds ratio; SE, standard error

For 2009, the intercept-only model showed an extremely low variation in multiple partnerships across neighbourhoods. Model-4, however, showed that being females and residing in neighbourhoods with high comprehensive knowledge gave lower odds of having multiple partners (Table 4).

### Condom use at last premarital sex

Condom use at last premarital sex generally remained stable among men between 2000 and 2009, whereas it decreased non-significantly among women. Significant falling trends occurred among women in neighbourhoods with medium residential stability and low comprehensive knowledge of HIV (women) (Table 5).

**Table 5:** Condom use trends among young people (15–24 years) by gender and by survey year (percentage).

		Female					Male				
		2000	2003	2005	2009	*p*	2000	2003	2005	2009	*p*
**Dependent variable**											
Condom use at last pre-marital sex		40.4	35.4	29.9	33.3	*0.247*	37.5	39.7	37.5	38.9	*0.908*
**Independent variables**											
***Individual variables***											
Age at last birthday											
	15–19	41.4	35.8	24.3	34.3	*0.274*	35.4	35.0	35.6	31.8	*0.625*
	20–24	38.2	34.7	38.3	32.0	*0.597*	39.7	43.1	38.8	45.8	*0.511*
Highest level of school attended											
	None/Primary	33.9	18.0	16.4	27.5	*0.378*	27.7	26.2	27.5	30.6	*0.643*
	Secondary/Higher	48.8	48.1	43.9	36.8	*0.125*	49.2	56.1	50.0	43.7	*0.223*
Employment											
	Not employed	47.2	32.3	28.4	33.7	*0.193*	44.9	42.2	39.6	36.6	*0.217*
	Employed	32.7	37.7	35.5	33.3	*0.994*	29.9	38.1	34.3	43.9	*0.184*
Religion											
	Catholic Christians	57.1	33.3	35.0	54.5	*0.898*	44.7	42.6	44.2	31.3	*0.285*
	Protestant Christians	35.8	35.7	29.3	28.7	*0.228*	34.3	38.6	35.6	39.9	*0.462*
Residence											
	Rural	32.3	29.3	16.7	22.1	*0.108*	31.3	30.2	30.9	31.4	*0.947*
	Urban	52.5	43.3	50.0	50.0	*0.963*	48.9	55.7	52.3	52.6	*0.873*
***Neighbourhood variables***											
Educational attainment											
	Low	46.7	40.6	52.9	46.6	*0.775*	47.4	43.2	44.3	41.3	*0.538*
	Medium	37.2	33.9	16.9	23.4	*0.068*	29.0	35.6	36.5	31.9	*0.776*
	High	31.3	21.1	29.2	8.3	*0.245*	37.9	41.7	24.2	48.6	*0.483*
Labour force participation											
	Low	51.2	32.8	30.0	25.0	***0.013***	54.5	51.5	40.6	39.0	***0.036***
	Medium	36.2	41.8	34.1	40.8	*0.772*	30.4	34.5	36.1	44.6	*0.073*
	High	21.4	28.0	18.8	35.0	*0.427*	22.6	25.0	29.2	26.7	*0.682*
Residential stability											
	Low	52.1	35.5	46.4	45.3	*0.857*	41.8	42.3	43.2	39.4	*0.784*
	Medium	31.8	40.4	14.8	17.1	***0.026***	37.9	40.0	34.1	37.3	*0.811*
	High	25.0	18.8	14.3	34.8	*0.357*	26.7	32.6	31.0	42.5	*0.166*
Comprehensive knowledge											
	Low	43.6	28.8	19.6	22.9	***0.015***	29.2	31.5	33.3	35.1	*0.419*
	Medium	34.3	33.3	36.4	25.0	*0.359*	46.8	38.5	44.0	40.3	*0.624*
	High	42.9	58.3	42.9	68.0	*0.176*	38.1	65.8	35.0	45.0	*0.652*

***P = *** p-values of the chi^2^ linear-by-linear trend test; highlighted figures are significant at p<0.05

Model-1 for 2000 showed that condom use varied across neighbourhoods, with a neighbourhood difference of 18%. The addition of individual variables (model-2) explained ∼10% of the variance in condom use among young people. The difference across neighbourhoods was reduced to 9%. Model-3, with neighbourhood variables only, explained 15% of the variance and the difference across neighbourhoods was reduced to 5%. Model-4 explained ∼18% of the variance, whereas the difference across neighbourhoods was reduced to 3%. The background variables that were significantly associated with lower odds of condom use in model-4 were being a protestant Christian and residing in neighbourhoods with lower labour force participation. Young people residing in neighbourhoods with high educational attainment had higher odds of condom use (Table 6).

**Table 6:** Multilevel logistic regression models for condom use at last pre-marital sex among young people aged 15-24 years in Zambia, in 2000 and 2009.

Dependent variables		Condom use at last premarital sex 2000				Condom use at last premarital sex 2009			
		Multivariate				Multivariate			
		Model 1	Model 2	Model 3	Model 4	Model 1	Model 2	Model 3	Model 4
			AOR	AOR	AOR		AOR	AOR	AOR
Fixed effects		(95% Cl)	(95% Cl)	(95% Cl)	(95% Cl)	(95% Cl)	(95% Cl)	(95% Cl)	(95% Cl)
***Individual variables***									
Sex									
	Male		1.00		1.00		1.00		1.00
	Female		1.17 (0.64–2.12)		1.22 (0.68–2.19)		0.71 (0.42–1.21)		0.73 (0.43–1.24)
Age			1.02 (0.89–1.17)		0.99 (0.86–1.13)		0.99 (0.89–1.10)		0.98 (0.88–1.09)
Highest level of school attended									
	None/Primary		1.00		1.00		1.00		1.00
	Secondary/Higher		1.61 (0.84–3.09)		1.61 (0.84–3.09)		1.29 (0.73–2.28)		1.32 (0.74–2.34)
Employment									
	Not employed		1.00		1.00		1.00		1.00
	Employed		0.67 (0.34–1.32)		0.92 (0.44–1.90)		1.26 (0.69–2.29)		1.22 (0.67–2.21)
Religion									
	Catholic Christians		1.00		1.00		1.00		1.00
	Protestant Christians		0.53 (0.27–1.05)^*^		0.53 (0.28–1.03)^*^		0.81 (0.43–1.55)		0.89 (0.46–1.70)
Residence									
	Rural		1.00		1.00		1.00		1.00
	Urban		2.03 (0.98–4.21)^*^		1.05 (0.47–2.34)		2.70 (1.51–4.83)^**^		3.43 (1.63–7.20)^**^
***Neighbourhood variables^β^***									
	Educational attainment			1.53 (1.06–2.19)^**^	1.58 (1.08–2.30)^**^			0.87 (0.65–1.17)	0.99 (0.74–1.35)
	Labour force participation			0.42 (0.27–0.63)^***^	0.45 (0.26–0.76)^**^			1.10 (0.78–1.53)	1.24 (0.87–1.75)
	Residential stability			0.88 (0.63–1.24)	0.87 (0.62–1.22)			1.08 (0.81–1.43)	1.48 (1.07–2.05)^**^
	Comprehensive knowledge			1.01 (0.73–1.38)	0.98 (0.72–1.35)			1.55 (1.15–2.08)^**^	1.39 (1.04–1.87)^**^
**Fixed –effect model test**									
	Wald *x^2^*		15.43^**^	20.49^**^	26.32^**^		16.05^**^	11.70^**^	25.17^**^
	Degree of freedom		6	4	10		6	4	10
**Unexplained neighbourhood-level variance (SE)**		0.701 (0.41)^**^	0.369 (0.31)^**^	0.194 (0.25)	0.110 (0.24)	0.471 (0.38)^**^	0.087 (0.26)	0.118 (0.29)	2.91e–16
**Model statistics**									
	Explained variance (R^2^)		0.102	0.148	0.184		0.073	0.059	
	Unexplained neighbourhood-level variance as	0.18	0.090	0.047	0.026	0.12	0.024	0.033	
	a proportion of total variance								
	Unexplained individual level variance as a		0.808	0.804	0.790		0.903	0.909	
	proportion of total variance								
	Log likelihood	−168.81	−153.60	−158.53	−147.87	−194.71	−181.36	−189.14	−176.71
	Log likelihood test^δ^		0.014	0.001	0.002		0.013	0.025	0.004

Highlighted figures statistically significant at the following points: ^*^P<0.10; ^**^P<0.05; ^***^P<0.01; ^β^Neighbourhood variables interpreted as OR per unit increase; **^δ^**Tests whether adding individual and neighbourhood variables to model-1 significantly improves the fit of the model (P<0.05); Abbreviations:R^2^, explained variance; Cl, confidence interval; AOR, adjusted odds ratio; SE, standard error

The intercept-only model in 2009 showed that condom use varied across neighbourhoods, with a neighbourhood difference of 12%. The inclusion of individual variables in model-2 and neighbourhood variables in model-3 reduced the differences across neighbourhoods to 2 and 3%, respectively. However, individual variables explained ∼7% of the variance in condom use, while neighbourhood variables explained ∼6% of the variance. The addition of both individual and neighbourhood variables in model-4 reduced the difference between neighbourhoods to an extremely low level. Young people residing in neighbourhoods with high residential stability and high comprehensive knowledge of HIV had higher odds of condom use (Table 6).

## Discussion

Sexual behaviour surveys based on the nationally representative samples of young adults in Zambia showed a decline in premarital sex for both females and males between 2000 and 2009. A substantially downward trend of multiple partnerships occurred among young men, whereas the proportion with multiple partnerships among young women during this period was relatively low and stable. However, condom use at last premarital sex remained relatively stable among males over the years, but decreased (although not significantly) among females. These findings have also been reported in the 2009 Zambia Sexual Behaviour Survey report [34]. In this study, we have also shown that the decline in trends also occurred in most sub-groups of young people for premarital sex (both men and women) and multiple partnerships (men only). Multilevel analysis further showed that both individual and neighbourhood variables influenced young people's sexual risk behaviour in both 2000 and 2009.

### Trends in sexual risk behaviours

It is clear from our findings that the downward trends of premarital sex provide a good indication that young people are postponing their sexual debut in Zambia. The decline does not seem to be due to an increased rate of early marriage, since the proportion of young people who had never been married decreased. Combined with decreasing trends of multiple partnerships among young men, this suggests positive sexual behaviour changes among young people in Zambia. The behavioural changes could have contributed to the decline in HIV incidence in Zambia since 2001. It is possible that the decline in premarital sex and multiple partnerships is a sign that HIV prevention campaigns promoting abstinence and faithfulness to one partner could have had an impact. It is also likely however that young people's personal experience of the effects of the HIV epidemic have led to these behavioural changes [43].

One of the targets set in the millennium development goal-6 was to increase condom use during high-risk sex among young people to > 90% by 2015 [44]. Despite extensive condom promotion in Zambia, condom use at high risk sex is currently estimated to be 40% [34]. In fact, condom use at last premarital sex has remained consistently low since 2000. The evidence indicates that there are many social barriers to condom use, for example, the influence of the church. In a study analysing data from the 2003 Zambia Sexual Behaviour Survey found that more than two-thirds of the participants believed that condoms promoted promiscuity [25]. Other factors affecting condom use include lack of money to buy condoms, the infrequent supply of condoms, long distance to outlets, stigma, lack of knowledge and gender inequality [25], [45], [46].

### Associations between predictors and sexual risk behaviours

Findings from the multilevel analyses offer insights into possible neighbourhood contextual effects on premarital sex, multiple partnerships and condom use among young people in Zambia. We have shown that the addition of individual factors to the intercept-only model substantially reduced, or in some cases increased, the difference between neighbourhoods. Increase in the total unexplained neighbourhood variance of premarital sex and multiple partnerships compared to the intercept-only model's variance when the individual-level factors were added to the models may be explained by the fact that the formula used assumes simple random sampling, so when grouped data are entered into the model, the lower level variance tends to increase [40]. However, several individual factors were associated with increased risk of premarital sex and multiple partnerships. In contrast, the condom use indicator had very few statistically significant associations with the individual factors; the probable reason could be that the small sample size within the clusters might have reduced the power to detect significant associations with the outcome factors.

This study also found that urban residence was significantly associated with lower likelihood of premarital sex and a higher likelihood of condom use in both 2000 and 2009, suggesting reduction in sexual risk behaviour among young urban males and females. These findings support the observation that HIV prevention efforts have been more intensive in urban settings. An example of this can be drawn from the condom distribution campaigns in Zambia, where an estimated 14 million condoms were distributed in 2008, with the majority of the distribution outlets being located in urban settings [8]. Furthermore, due to the high prevalence of HIV in urban areas, urban residents are more likely to have greater personal experience of HIV/AIDS and may perceive themselves at higher risk of infection and thus be more motivated to avoid risk taking.

To check for independent neighbourhood effects, we entered neighbourhood factors into the intercept-only models. This resulted in a substantial decline in the unexplained variance of premarital sex (in 2000) and condom use at last premarital sex (in 2000 and 2009) at the neighbourhood-level. This suggests that neighbourhood variables have a considerable influence on the sexual risk behaviour indicators. To assess the neighbourhood-level variance explained by these variables, we initially considered the formula suggested by Raudenbush and Bryk [42]. We could explain 62% of the variance of young people's premarital sex in 2000, and 72 and 75% of the variance of young people's condom use in 2000 and 2009, respectively, which were high proportions. For this particular formula, the explained intercept variance is based on a comparison of the intercept-only model and the model without random slopes, and thus has the two weaknesses pointed out by Hox [42]. First, it is possible to arrive at the conclusion that specific factors contribute negatively to explained variance, and second in random slope models the estimated variances depend on the scale of the explanatory factors [42]. Conversely, using the formula suggested by Snijders and Bosker [41], their explained variance (R^2^) is a proportion of the total variance, because first level variables in principle, can explain all variation, including that at the second level [42]. This formula removes the spurious increase in variance when variables are added to the intercept-only model, thus avoiding the negatively explained variance.

Neighbourhood residential stability was a statistically significant predictor of both premarital sex and condom use (in 2000 and 2009, respectively). We argue that this predictor can be used as a proxy for social capital [38], since the longer people stay in the same neighbourhood, the more they are likely to interact with each other, build trust and develop a strong community solidarity [47]. This social cohesion has an impact on young people's sexual risk behaviour [48], [49]. Our study supports this, since we found that, per unit of increase in the variance of average length of residence in the neighbourhoods, the odds of premarital sex decreased and that of condom use increased among young people. High neighbourhood residential stability is likely to increase social control over young people's behaviour, thus limiting their perceived opportunities to engage in premarital sex [38]. Other studies have found that young people in communities with a high social capital have a higher tendency to delay their sex debut and use protection during sexual intercourse [49].

Another striking neighbourhood predictor of lower risk of premarital sex and multiple partnerships, and higher condom use in our study, is comprehensive knowledge of HIV. The 2007 Zambia Demographic and Health Survey indicated that >90% of Zambia's population were aware of HIV, but only 37% of young men and 34% of women had comprehensive knowledge of HIV transmission [29]. This is way below the 95% target set in the 2001 UNGASS Declaration [1]. Although knowledge alone is not enough to change behaviour [50], these findings support earlier evidence that an increase of comprehensive knowledge of HIV reduces the risk of new infections, especially among young people [1], [51].

Neighbourhood educational attainment may be viewed as a proxy for neighbourhood socio-economic position. High neighbourhood socio-economic position has been found to reduce the risk of premarital sex [21]. In contrast, another study found that women in communities with a higher proportion of educated community members had significantly greater odds of engaging in premarital sex than those with lower proportions of educated people [38]. Our study however found that neighbourhood educational attainment was only significantly associated with condom use in 2000. At the individual level, young educated people tended to have higher odds of engaging in premarital sex (non-significant association) and having multiple partners. This may reflect that young people with high education may feel more liberated from social norms that restrict premarital sex than their less educated peers, possibly because they are absent from home attending school. However, these findings are surprising considering that a number of studies in Zambia have shown a clearer decrease in sexual risk behaviour [5] and HIV prevalence among educated young people than the less educated since the late 1990s [2], [3], [5].

The different aspects of average neighbourhood employment, for example labour force participation, the prevalence of full time employment, and employment opportunities, have been used as proxies of the ability of adults to supervise young people's behaviour [20].This is because neighbourhoods with high employment rates usually comprise people who are highly educated, and they are often characterised by family stability, high levels of parental authority and control, availability of parental time and supervision, and increased communication between the young people and their parents [52]. We found that neighbourhood labour force participation was associated with more premarital sex and less condom use in 2000, but neighbourhood labour force participation was associated with less premarital sex in 2009. The findings may reflect that the questions regarding employment status in the early and later surveys measured different aspects of employment, which may have caused the differences seen between the early and later surveys in the regression analyses rather than this being a major change in the association between sexual behaviour and employment status.

To measure the mediating effects of individual and neighbourhood factors, they were entered into the same model. For example, the full multivariate model for premarital sex in 2000 showed that neighbourhood labour force participation became statistically insignificance. It is possible that neighbourhood labour force participation was a confounder in model-3 and that the important association was between the individual-level employment status and premarital sex. Furthermore, neighbourhood comprehensive knowledge in 2000 lost its statistical significance in the full multivariate models for multiple partnerships, which might have been a result of partial confounding of the effect of rural-urban residence variable. Urban-rural residence seemed to be a confounder in relation to condom use at last premarital sex in 2000, since the significant association seen in model-2 disappeared when adjusted for neighbourhood educational attainment and neighbourhood labour force participation. In contrast, after the urban-rural residence variable was added to the full multivariate models in 2009, neighbourhood comprehensive knowledge became a significant predictor of multiple partnerships, neighbourhood residential stability became significantly associated with condom use at last premarital sex, and neighbourhood labour force participation became a significant predictor of premarital sex. In these cases urban/rural residence seems to have functioned as a negative confounder that distorted the true association toward the null.

### Limitations of the study

The results of this study should be considered with some caution. For instance, it was based on self-reported sexual behaviour data, i.e. subject to bias. Some studies have found considerable errors in self-reporting of sexual activity, for example, males over-reporting and females under-reporting their sexual activities [53], [54], [55]. This may also have been the case in our data since men reported on average many more partners than young women. Young men are subtly expected in most cultures to display high heterosexual activity, whereas young women are expected to be chaste [15], [56]. Therefore, the difference between the reports of men and women may be due to men overstating their reported number of sexual partners [57].

Furthermore, it is possible that using face-to-face interviews to gather this type of information may have contributed to reporting errors as people might not reveal all their sex life details to a stranger. If the magnitude of the bias has changed over time, this would have affected our trend estimates. The cross-sectional nature of the data limits our ability to draw causal inferences about the associations we have found. Moreover, the use of repeated cross-sectional studies conducted in different clusters, with different households, to estimate trends would affect estimates if there had been a major population shift over the years being analysed. However, such major shifts do not seem to have occurred. The number of neighbourhood and individual-level variables used in this study was restricted by the changes in the questionnaires over the years. Large proportions of the total variance of the three indicators were not explained by the individual or the neighbourhood factors, and the explanation for this could be that we did not include all the important factors. We have only assessed trends in 3 sexual behaviour indicators, and there may be other important aspects of changes in sexual behaviour among young people that might have been missed [10], [11]. The small sample size, especially for the analysis of multiple partnerships and condom use, might have resulted in low power to detect differences at both individual and neighbourhood levels.

### Study Implications

To be useful to policy makers in Zambia, future neighbourhood research on the effect of neighbourhood on sexual risk behaviour should not only be focused on *whether* neighbourhood factors influence sexual behaviour but begin to tackle critical questions of *how* these factors affect sexual risk behaviour. Furthermore, there is need for these future studies to be based on theory at design stage, for example, the Social Capital theory or the Ecological model of human development, so that the relationships found can be properly explained.

### Conclusion

This study showed that premarital sex and multiple partnerships have been decreasing over the years but condom use has remained almost stable since 2000. In addition, the present study showed that both individual and neighbourhood contextual factors played a considerable role in influencing the sexual risk behaviours of young people.

## Supporting Information

Table S1 **Operational Definitions of the Variables.**
(DOC)

Table S2 **Descriptive statistics of young people aged 15–24 years stratified by survey year, in ZSBS 2000–2009 (percentage).**
(DOC)

Table S3 **Premarital sex trends among young people (15–24 years) from 2000 to 2009 (in percentage).**
(DOC)

Table S4 **Multiple Partnerships trends among young people (15–24 years) from 2000 to 2009 (in percentage).**
(DOC)

Table S5 **Condom use at last sex trends among young people (15–24 years) from 2000 to 2009 (in percentage).**
(DOC)
