# Correction: Decline in Sexual Risk Behaviours among Young People in Zambia (2000–2009): Do Neighbourhood Contextual Effects Play a Role?

**DOI:** 10.1371/annotation/c9ea65f1-b9ff-41e3-998d-e7bdb722ae8c

**Published:** 2014-01-06

**Authors:** Nkomba Kayeyi, Knut Fylkesnes, Nora Wiium, Ingvild F. Sandøy

Tables S3, S4 and S5 contain incorrect information. Please see the corrected Tables below.

Table S3: http://plosone.org/corrections/pone.0064881.s003.cn.doc

Table S4: http://plosone.org/corrections/pone.0064881.s004.cn.doc

Table S5: http://plosone.org/corrections/pone.0064881.s005.cn.doc

