# Supplementary material for: Decline in Sexual Risk Behaviours among Young People in Zambia (2000–2009): Do Neighbourhood Contextual Effects Play a Role?
Source: PLoS One. 2013 May 23;8(5):e64881. doi: 10.1371/journal.pone.0064881 (PMC3662790; doi:10.1371/journal.pone.0064881)
Supplement: Table S1 — Operational Definitions of the Variables. (DOC) [file pone.0064881.s001.doc]

**Table S1. Operational Definitions of the Variables**

| **Variable** | **Operational Definitions** | **Responses** |
| --- | --- | --- |
| ***Behaviour Variable*** |  |  |
| Premarital sex* | Percentage of young single people (aged 15-24 years) who had | 1. No |
|  | sex in the last 12 months of all single people surveyed | 1. Yes |
| Multiple partners* | Percentage of young people (aged 15-24 years) who had two or | 1. One |
|  | more sexual partners last year, of all young people surveyed | 1. Two or more |
| Condom use at last sex^*^ | Percentage of young single people who used a condom at last | 1. No |
|  | sex, of all young single sexually active people surveyed | 1. Yes |
| ***Individual variables*** |  |  |
| Sex | Male or female | 1. Male |
|  |  | 1. Female |
|  |  |  |
| Age at survey | Age at last birthday | 15 – 24 years |
|  |  |  |
| Ever Married | Percentage of young people who have ever been married | 1. Never |
|  |  | 1. Married |
| Education | Highest level of schooling attained | 1. None/primary |
|  |  | 1. Secondary/Higher |
| Employment status^**^ | Current employment status | 1. Not employed |
|  |  | 1. Employed |
| Religion | Religious affiliation | 1. Catholic |
|  |  | 1. Protestant |
| Place of residence | Current residence place | 1. Rural |
|  |  | 1. Urban |
| ***Neighbourhood variables*** |  |  |
| Educational attainment | Average educational attainment among people in the |  |
|  | neighbourhoods |  |
|  |  |  |
| Labour force participation^**^ | Average labour force participation in the neighbourhoods |  |
|  |  |  |
| Residential stability | Average years of stay in the neighbourhood |  |
|  |  |  |
| Comprehensive HIV knowledge | Average number of people in the neighbourhood who |  |
|  | correctly answered the questions below: |  |
|  | - Can a person who looks healthy be infected with HIV? |  |
|  | - Can people reduce their chances of getting the AIDS virus by |  |
|  | using a condom correctly every time they have sex? |  |
|  | - Can people reduce their chances of getting the AIDS virus by |  |
|  | having only one sex partner who has no other partners? |  |
|  | - Can HIV be transmitted by mosquitoes? |  |
|  | - Can HIV be transmitted by witchcraft? |  |

*HIV/AIDS survey indicators defined by UNAIDS; all of them were considered as dependent variables. ^**^In the 2000 and 2003 surveys, the question asked for the employment variable was: “what kind of work do you mainly do?” and in 2005 and 2009 surveys the question used in this study was: “are you currently working?” but responses to both questions were categorised as “employed” and “non-employed”.
