# Supplementary material for: Decline in Sexual Risk Behaviours among Young People in Zambia (2000–2009): Do Neighbourhood Contextual Effects Play a Role?
Source: PLoS One. 2013 May 23;8(5):e64881. doi: 10.1371/journal.pone.0064881 (PMC3662790; doi:10.1371/journal.pone.0064881)
Supplement: Table S2 — Descriptive statistics of young people aged 15–24 years stratified by survey year, in ZSBS 2000–2009 (percentage). (DOC) [file pone.0064881.s002.doc]

**Table S2.** Descriptive statistics of young people aged 15-24 years stratified by survey year, in ZSBS 2000–2009 (percentage)

| Year | | **2000** | **2003** | **2005** | **2009** |
| --- | --- | --- | --- | --- | --- |
|  | |  |  |  |  |
| Sample population (n) | | 1376 | 1835 | 1695 | 1594 |
| **Dependent variables** | |  |  |  |  |
| Pre-marital sex | |  |  |  |  |
|  | No | 49.2 | 46.4 | 52.9 | 57.6 |
|  | Yes | 50.8 | 53.6 | 47.1 | 42.4 |
| Multiple partners last 12 months | |  |  |  |  |
|  | 1 partner | 88.7 | 90.2 | 91.1 | 93.7 |
|  | 2 or more partners | 11.3 | 9.8 | 8.9 | 6.3 |
|  | Condom use at last premarital sex |  |  |  |  |
|  | No | 61.4 | 61.9 | 65.2 | 63.1 |
|  | Yes | 38.6 | 38.1 | 34.8 | 36.9 |
| **Independent variables** | |  |  |  |  |
| ***Individual variables*** | |  |  |  |  |
| Age at last birthday | |  |  |  |  |
|  | Mean age - years (S.D.) | 19.36 (2.80) | 19.37 (2.74) | 19.42 (2,82) | 19.23 (2,88) |
| Gender | |  |  |  |  |
|  | Male | 40.5 | 45.0 | 44.5 | 45.9 |
|  | Female | 59.5 | 55.0 | 55.5 | 54.1 |
| Ever married | |  |  |  |  |
|  | Never | 61.5 | 65.7 | 63.8 | 72.8 |
|  | Married | 38.5 | 34.3 | 36.2 | 27.2 |
| Highest level of school attended | |  |  |  |  |
|  | None/Primary | 61.8 | 58.6 | 57.4 | 48.7 |
|  | Secondary/Higher | 38.2 | 41.4 | 42.6 | 51.3 |
| Employment | |  |  |  |  |
|  | Not employed | 47.5 | 37.0 | 70.8 | 78.2 |
|  | Employed | 52.5 | 63.0 | 29.2 | 21.8 |
| Religion | |  |  |  |  |
|  | Catholic Christians | 23.9 | 24.2 | 22.3 | 18.8 |
|  | Protestant Christians | 76.1 | 75.8 | 77.7 | 81.2 |
| Residence | |  |  |  |  |
|  | Rural | 59.7 | 61.1 | 63.9 | 59.5 |
|  | Urban | 40.3 | 38.9 | 36.1 | 40.5 |
| ***Neighbourhood variables*** | |  |  |  |  |
| Educational attainment - mean (S.D.) | | 4.37 (0.60) | 3.91 (0.57) | 4.50 (0.86) | 3.94 (0.55) |
|  | (Min – Max) | (3.00 – 5.96) | (2.53 – 5.50) | (2.58 – 6.91) | (2.28 – 0.86) |
| Labour force participation - mean (S.D.) | | 0.72 (0.18) | 0.80 (0.12) | 0.47 (0.16) | 0.44 (0.14) |
|  | (Min – Max) | (0.36 – 1.00) | (0.50 – 1.00) | (0.13 – 0.91) | (0.10 – 0.82) |
| Residential stability - mean (S.D.) | | 11.88 (3.75) | 12.27 (4.0.3) | 12.25 (4.26) | 12.62 (3.94) |
|  | (Min – Max) | (4.40 – 20.4) | (4.08 – 24.4) | (3.71 – 22.03) | (4.98 – 22.0.5) |
| Comprehensive knowledge - mean (S.D.) | | 0.12 (0.08) | 0.33 (0.17) | 0.44 (0.21) | 0.43 (0.18) |
|  | (Min – Max) | (0.0 – 0.35) | (0.0 – 0.70) | (0.02 – 0.86) | (0.08 – 0.85) |

All neighbourhood variables were analysed as continuous variable; n, sample population; S.D., Standard deviation; Min – Max, minimum and maximum
