# Supplementary material for: Decline in Sexual Risk Behaviours among Young People in Zambia (2000–2009): Do Neighbourhood Contextual Effects Play a Role?
Source: PLoS One. 2013 May 23;8(5):e64881. doi: 10.1371/journal.pone.0064881 (PMC3662790; doi:10.1371/journal.pone.0064881)
Supplement: Table S3 — Premarital sex trends among young people (15–24 years) from 2000 to 2009 (in percentage). (DOC) [file pone.0064881.s003.doc]

**Table S3.** Premarital sex trends among young people (15-24 years) from 2000 to 2009 (in percentage)

| Year | | **2000** | **2003** | **2005** | **2009** |
| --- | --- | --- | --- | --- | --- |
| **Dependent variables** | |  |  |  |  |
|  | Pre-marital sex - n (%) | 841 (50.8) | 1205 (53.6) | 1080 (47.1) | 1159 (42.4) |
| **Independent variables** | |  |  |  |  |
| ***Individual variables*** | |  |  |  |  |
| Age at last birthday | |  |  |  |  |
|  | Mean age - years (S.D.) | 19,35 (2.55) | 19.37 (2.39) | 19.56 (2.49) | 19.73 (2.52) |
| Gender | |  |  |  |  |
|  | Male | 239 (54.4) | 403 (57.5) | 321 (52.5) | 294 (45.1) |
|  | Female | 188 (46.8) | 243 (48.2) | 188 (40.2) | 197 (38.9) |
| Highest level of school attended | |  |  |  |  |
|  | None/Primary | 214 (48.4) | 309 (50.8) | 247 (49.5) | 169 (34.9) |
|  | Secondary/Higher | 213 (53.5) | 336 (56.4) | 261 (45.1) | 322 (47.8) |
| Employment | |  |  |  |  |
|  | Not employed | 210 (40.5) | 289 (46.2) | 344 (40.3) | 370 (37.9) |
|  | Employed | 217 (67.2) | 357 (61.7) | 155 (73.1) | 119 (67.2) |
| Religion | |  |  |  |  |
|  | Catholic Christians | 97 (46.2) | 163 (55.3) | 124 (49.2) | 94 (40.9) |
|  | Protestant Christians | 309 (52.6) | 482 (53.1) | 383 (46.8) | 390 (42.6) |
| Residence | |  |  |  |  |
|  | Rural | 240 (55.4) | 364 (54.6) | 301 (50.7) | 285 (44.4) |
|  | Urban | 187 (45.8) | 282 (52.4) | 208 (42.8) | 206 (39.8) |
| ***Neighbourhood variables*** | |  |  |  |  |
| Educational attainment - mean (S.D.) | | 4.42 (0.60) | 3.94 (0.56) | 4.56 (0.85) | 3.96 (0.58) |
|  | (Min – Max) | (3.00-5.96) | (2.53-5.50) | (2.58-6.91) | (2.28-5.42) |
| Labour force participation - mean (S.D.) | | 0.74 (0.17) | 0.81 (0.12) | 0.48 (0.17) | 0.43 (0.12) |
|  | (Min – Max) | (0.36-1.00) | (0.50-1.00) | (0.13-0.91) | (0.10-0.82) |
| Residential stability - mean (S.D.) | | 12.01 (3.79) | 12.17 (4.04) | 12.54 (4.24) | 12.72 (4.02) |
|  | (Min – Max) | (4.40-20.40) | (4.08-24.38) | (3.71-22.03) | (4.98-22.05) |
| Comprehensive knowledge - mean (S.D.) | | 0.11 (0.07) | 0.33 (0.18) | 0.42 (0.22) | 0.42 (0.18) |
|  | (Min – Max) | (0.00-0.35) | (0.00-0.70) | (0.02-0.86) | (0.08-0.85) |

All neighbourhood variables were analysed as continuous variable; n, sample population; S.D., Standard deviation; Min – Max, minimum and maximum
