# Supplementary material for: Decline in Sexual Risk Behaviours among Young People in Zambia (2000–2009): Do Neighbourhood Contextual Effects Play a Role?
Source: PLoS One. 2013 May 23;8(5):e64881. doi: 10.1371/journal.pone.0064881 (PMC3662790; doi:10.1371/journal.pone.0064881)
Supplement: Table S4 — Multiple Partnerships trends among young people (15–24 years) from 2000 to 2009 (in percentage). (DOC) [file pone.0064881.s004.doc]

**Table S4.** Multiple Partnerships trends among young people (15-24 years) from 2000 to 2009 (in percentage)

| Year | | **2000** | **2003** | **2005** | **2009** |
| --- | --- | --- | --- | --- | --- |
| **Dependent variables** | |  |  |  |  |
|  | Multiple partnerships - n (%) | 759 (11.3) | 971 (9.8) | 894 (8.9) | 702 (6.3) |
| **Independent variables** | |  |  |  |  |
| ***Individual variables*** | |  |  |  |  |
| Age at last birthday | |  |  |  |  |
|  | Mean age - years (S.D.) | 20.40 (2.59) | 20.84 (2.33) | 20.39 (2.84) | 20.61 (2.56) |
| Gender | |  |  |  |  |
|  | Male | 69 (25.8) | 69 (19.3) | 52 (15.1) | 35 (14.1) |
|  | Female | 17 (3.5) | 26 (4.2) | 28 (5.1) | 9 (2.0) |
| Ever married | |  |  |  |  |
|  | Never | 51 (19.5) | 58 (15.2) | 48 (15.2) | 28 (9.6) |
|  | Married | 34 (6.9) | 37 (6.3) | 32 (5.5) | 16 (3.9) |
| Highest level of school attended | |  |  |  |  |
|  | None/Primary | 46 (8.8) | 55 (8.7) | 51 (8.3) | 17 (4.4) |
|  | Secondary/Higher | 40 (17.1) | 40 (11.8) | 28 (10.1) | 27 (8.5) |
| Employment | |  |  |  |  |
|  | Not employed | 23 (8.9) | 25 (12.6) | 53 (10.0) | 29 (6.2) |
|  | Employed | 63 (12.5) | 70 (9.1) | 26 (7.4) | 15 (6.5) |
| Religion | |  |  |  |  |
|  | Catholic Christians | 17 (11.2) | 15 (6.5) | 8 (4.2) | 8 (7.0) |
|  | Protestant Christians | 63 (11.2) | 80 (10.8) | 72 (10.3) | 36 (6.2) |
| Residence | |  |  |  |  |
|  | Rural | 63 (11.8) | 55 (8.3) | 65 (9.8) | 28 (5.9) |
|  | Urban | 23 (10.1) | 40 (13.1) | 15 (6.5) | 16 (7.0) |
| ***Neighbourhood variables*** | |  |  |  |  |
| Educational attainment - mean (S.D.) | | 4.35 (0.60) | 3.86 (0.59) | 4.33 (0.78) | 3.91 (0.55) |
|  | (Min – Max) | (3.00-5.96) | (2.53-5.08) | (2.58-6.91) | (2.28-5.24) |
| Labour force participation - mean (S.D.) | | 0.68 (0.17) | 0.80 (0.12) | 0.45 (0.14) | 0.68 (0.42) |
|  | (Min – Max) | (0.36-0.98) | (0.50-1.00) | (0.13-0.88) | (0.10-0.68) |
| Residential stability - mean (S.D.) | | 10.99 (3.26) | 11.47 (4.01) | 11.53 (4.07) | 12.56 (4.29) |
|  | (Min – Max) | (4.40-20.40) | (4.08-21.80) | (3.71-19.63) | (4.98-21.79) |
| Comprehensive knowledge - mean (S.D.) | | 0.11 (0.08) | 0.34 (0.17) | 0.45 (0.22) | 0.44 (0.19) |
|  | (Min – Max) | (0.00-0.35) | (0.03-0.70) | (0.02-0.80) | (0.08-0.85) |

All neighbourhood variables were analysed as continuous variable; n, sample population; S.D., Standard deviation; Min – Max, minimum and maximum
