# Supplementary material for: Decline in Sexual Risk Behaviours among Young People in Zambia (2000–2009): Do Neighbourhood Contextual Effects Play a Role?
Source: PLoS One. 2013 May 23;8(5):e64881. doi: 10.1371/journal.pone.0064881 (PMC3662790; doi:10.1371/journal.pone.0064881)
Supplement: Table S5 — Condom use at last sex trends among young people (15–24 years) from 2000 to 2009 (in percentage). (DOC) [file pone.0064881.s005.doc]

**Table S5.** Condom use at last sex trends among young people (15-24 years) from 2000 to 2009 (in percentage)

| Year | | **2000** | **2003** | **2005** | **2009** |
| --- | --- | --- | --- | --- | --- |
| **Dependent variables** | |  |  |  |  |
|  | Condom use at last sex - n (%) | 259 (38.6) | 381 (38.1) | 325 (34.8) | 298 (36.9) |
| **Independent variables** | |  |  |  |  |
| ***Individual variables*** | |  |  |  |  |
| Age at last birthday | |  |  |  |  |
|  | Mean age - years (S.D.) | 19.30 (2.43) | 19.60 (2.27) | 19.80 (2.46) | 19.80 (2.62) |
| Gender | |  |  |  |  |
|  | Male | 58 (37.4) | 94 (39.7) | 78 (37.5) | 71 (39.2) |
|  | Female | 42 (40.4) | 51 (35.4) | 35 (29.9) | 39 (33.3) |
| Highest level of school attended | |  |  |  |  |
|  | None/Primary | 45 (30.8) | 45 (23.6) | 40 (23.3) | 36 (30.5) |
|  | Secondary/Higher | 55 (48.7) | 100 (52.9) | 73 (48.0) | 74 (41.1) |
| Employment | |  |  |  |  |
|  | Not employed | 58 (46.4) | 60 (38.5) | 76 (34.9) | 79 (35.7) |
|  | Employed | 42 (31.3) | 85 (37.8) | 34 (34.7) | 31 (40.8) |
| Religion | |  |  |  |  |
|  | Catholic Christians | 29 (49.2) | 36 (39.1) | 31 (40.8) | 22 (40.7) |
|  | Protestant Christians | 66 (34.9) | 108 (37.5) | 82 (33.2) | 84 (35.3) |
| Residence | |  |  |  |  |
|  | Rural | 54 (32.0) | 69 (29.7) | 54 (25.7) | 53 (28.3) |
|  | Urban | 46 (51.1) | 76 (51.0) | 59 (51.3) | 57 (51.8) |
| ***Neighbourhood variables*** | |  |  |  |  |
| Educational attainment - mean (S.D.) | | 4.28 (0.64) | 3.86 (0.60) | 4.45 (0.97) | 3.90 (0.55) |
|  | (Min – Max) | (3.00-5.96) | (2.53-5.08) | (2.58-6.91) | (2.28-5.42) |
| Labour force participation - mean (S.D.) | | 0.68 (0.17) | 0.79 (0.12) | 0.46 (0.14) | 0.42 (0.11) |
|  | (Min – Max) | (0.36-0.98) | (0.50-1.00) | (0.13-0.88) | (0.10-0.82) |
| Residential stability - mean (S.D.) | | 11.18 (3.28) | 11.17 (3.89) | 11.34 (4.50) | 12.07 (4.00) |
|  | (Min – Max) | (4.40-20.40) | (4.08-21.27) | (3.71-22.03) | (4.98-22.05) |
| Comprehensive knowledge - mean (S.D.) | | 0.11 (0.08) | 0.33 (0.17) | 0.47 (0.21) | 0.47 (0.19) |
|  | (Min – Max) | (0.00-0.35) | (0.06-0.66) | (0.02-0.84) | (0.08-0.85) |

All neighbourhood variables were analysed as continuous variable; n, sample population; S.D., Standard deviation; Min – Max, minimum and maximum
